# Suggestion for Determining Treatment Strategies in Dental Ethics

**DOI:** 10.1007/s11673-023-10310-2

**Published:** 2023-11-30

**Authors:** Szilárd D. Kovács

**Affiliations:** Institute of Behavioural Sciences, Semmelweis University, Budapest, 1089 Hungary

**Keywords:** Cosmetic dentistry, Dental ethics, Oral health, Treatment strategy, Value hierarchy

## Abstract

Contemporary medicine views health as the individual’s physical, mental, and social well-being. Oral health plays a crucial role in one’s well-being, as the oral cavity and its surrounding regions execute essential functions in verbal and nonverbal communication, sensing, digestion, and significantly contribute to aesthetic appearance. The multifaceted nature of the notion of oral health, as well as the patient’s needs and autonomous will result in various treatment options for the same oral state, favouring often contrasting ethical values and different aspects of oral health. The objective of this article is to suggest alternative treatment strategies in dentistry with respect to the following factors: extent of rehabilitation, preserving one’s anatomical structures, aesthetic outcome, number of sessions, patient autonomy. Additionally, this article describes the suggested treatment strategies in an ethical context and determines the conditions of their employment. The suggested treatment strategies are divided in two categories, *extensive treatment strategies* focusing on the patient’s entire craniofacial complex, while *specific treatment strategies* focus on specific paramount issues.

## Introduction

The members of a profession possess distinguished specified knowledge in their field and serve the public according to the professional ethics they adopt (Ozar, Sokol, and Patthoff 2018). In medical ethics Beauchamps’s and Childress’s four principles are commonly used to judge the ethical course of action: autonomy, nonmaleficence, beneficence, and justice (Kovács 2006; Beauchamp and Childress 1979). Ozar, Schiedermayer, and Siegler expanded these principles conforming it to dentistry and arranged them in the following hierarchy: 1) Life and health 2) Appropriate and pain-free oral functioning 3) Patient autonomy 4) Preferred practice values 5) Aesthetic values 6) Cost 7) External considerations, for which the specifically mentioned examples are the patient’s habits and circumstances, resource management, the agent aiding decision-making in case of patients with compromised autonomy, public interests, and the dentist’s additional responsibilities beside their occupation (Ozar, Schiedermayer, and Siegler 1988). Eventually the Central Practice Values of dentistry were created by Ozar and Sokol, where the previous ranking of values was modified as follows: 1) The Patient’s Life and General Health 2) The Patient’s Oral Health 3) The Patient’s Autonomy 4) The Dentist’s Preferred Patterns of Practice 5) Aesthetic Values 6) Efficiency in the Use of Professional Resources (Ozar, Sokol, and Patthoff 2018). Rule and Veatch argue that this hierarchy of values is flawed because individual dentists and the public might rank these values differently. Moreover, they expressed their doubt whether efficiency itself is in essence a value (Rule and Veatch 2004).

In the described sequences the top-ranking values are health, oral health, and autonomy, which need to be defined for the purposes of this article. The widely accepted definition of health is found in the constitution of the World Health Organization as one’s state of physical, social, and mental well-being (Larsen 2022). Furthermore, the WHO also introduces a three-layered model of health experience: Impairment is described as the loss or abnormality of psychological, physiological, or anatomical structure or function. Disability is restriction in performing a function which is generally perceived to be normal. Handicap is restriction in fulfilling a normal role in the person’s social context. It should be noted, that wearing dental prosthesis is specified as a subcategory of impairments (World Health Organization 1980). Adhering to the biopsychosocial concept of health, the World Dentist Federation (FDI) determines oral health as the ability of the craniofacial complex to speak, smile, smell, taste, touch, chew, swallow, and express emotions without pain, discomfort, or disease (Glick, et al. 2016). Finally, the definition of autonomy is the individual’s ability to comprehend alternatives without internal or external restrictions and the ability to act according to the desired alternative (Kovács 2006).

Since oral health involves one’s own satisfaction and adequate function in a social setting per definition, it cannot be entirely detached from aesthetics, which was a low-ranking value in the proposed value hierarchies. This path of reasoning is supported by literature: aesthetic appearance is associated with career success, higher popularity, more erotic experience, higher self-confidence, increased social skills, and better academic performance (Langlois, et al. 2000) and positive social outcomes are also linked to oral aesthetics (Kershaw, Newton, and Williams 2008; Eli, Bar-Tal, and I. Kostovetzki 2001; Feng, Newton, and Robinson 2001). To stress the significance of aesthetic value, literature indicates that dentistry encounters a heightened interest from the public for cosmetic procedures (Spear and Kokich 2007). According to surveys 13 to 38 per cent of lay people have undergone vital tooth bleaching (Samorodnitzky-Naveh, Geiger, and Levin 2007) and dental practitioners report a monthly regularity in performing vital tooth bleaching (Lussier and Benigeri 2008).

In conclusion the previously elaborated fundamental principles and values outline a theoretic objective of dentistry to maintain or restore an oral state aligning to the individual’s needs. However, this leaves room for various viable treatment plans for the same patient.

Gerostomatology is a field in dentistry, where three strategies have been developed in treatment planning with alternative objectives. In principal geriatric patients present higher rates of edentulism and progression of chronic oral conditions, albeit their need for dental interventions frequently faces barriers such as comorbidities, difficulties in mobilization, or by the compromized mental state of the patient. Therefore the alternative treatment strategies in gerostomatology mainly differ in the number of interventions associated with their objective. The *restorative treatment plan* intends a complete morphological restoration. By contrast the *rationalizing treatment plan* aims to reduce the visits to merely carrying out the most beneficial interventions, with focus on performing interventions in advance, e.g., extracting teeth with hopeless prognosis regardless of symptoms or lack thereof. Ultimately the *postponing treatment plan* is implemented if the patient refuses treatment and treatment is postponed until the patient deliberately asks for it. The provided characteristic examples for postponing treatment are geriatric patients who refuse tooth extractions because they perceive edentulism as the loss of their youth or patients with odontophobia. In this division of treatment plans literature advises pursuing a *rationalising treatment plan* for geriatric patients if the patient gives consent to employ this strategy (Fejérdy, Nagy, and Orosz 2007).

This deduction based on the number of interventions conforms to the limits of extensive dental care oftentimes present in geriatric patients, while also respecting patient autonomy. As it is not the goal of this grouping to entertain significant features in treatment planning in general dentistry, it only partially distinguishes the nature of the interventions and how they contribute to oral health and especially to aesthetic value.

The objective of this article is to suggest viable treatment strategies in dentistry marked by the following parameters: extent of rehabilitation, preserving own anatomical structures, aesthetic value, and number of sessions. Further on this article aims to present these strategies in the context of their relation to fundamental values of medical ethics. The author of this article wishes to initialiate a series of theoretical studies within the bioethical and dental community to determine regularly applicable ethical guidelines in dentistry concerning alternative treatment options.

## Suggested Treatment Strategies in Dentistry

### Idealistic Strategy

The *idealistic strategy* is akin to gerostomatology’s restorative strategy (Fejérdy, Nagy, and Orosz 2007). Following this strategy means conceptualizing an ideal, textbook-based morphology of the craniofacial complex and a function adhering to perfect gnathological standards. These ideals may result from a populace’s average, or they may even differ from it, as orthodontically ideal occlusion described by Angle being the exception rather than the norm (Artun 2002). In this case reaching the preferred morphological outcome is the aim, even after a reasonable function is attained and the functional benefits of further interventions are non-existent or marginal, for example by removing clinical sign-free and symptom-free impacted teeth or replacing second molars of a patient with an otherwise functional occlusion, as exemplified in gerostomatology’s restorative strategy (Fejérdy, Nagy, and Orosz 2007). Aesthetic advancement inevitably follows morphological rehabilitation, however additional procedures are not adopted for further aesthetic improvement in this strategy. Thus, function ranks higher than aesthetics. Preserving tooth structure is as moderately important as aesthetics, hence an *idealistic treatment plan* would permit extracting teeth with less than mediocre prognosis to adequately replace them. To pursue this strategy, a complex treatment plan needs to be proposed with numerous visits, followed by a regular follow-up aiming preventive care.

The disadvantage of this strategy may be the overtreatment of the patient, where the risks of the medical intervention outweigh the benefits, therefore the well-intentioned intervention results in violating the principal of nonmaleficence. Hence in practice a *nearly idealistic treatment plan*, which raises standards to the highest achievable goal without overtreatment is an ethically acceptable treatment plan and should be presented to the patient, given there are no restraints in accomplishing it. Hereon the patient’s own set of values determine whether they prefer an extensive morphological restoration in multiple visits to the dental office, even if not all sessions improve oral function significantly.

### Functionalist Strategy

This strategy could be described as a reduced version of the *idealistic strategy*, in which it is deprived of its idealistic core. Hence it does not demand morphological restoration but rather focuses on clinical signs or symptoms of impaired oral function, restoring the impaired function, and seeking to maintain the achieved state through regular follow-ups. Morphological restoration and preserving one’s dental tissues are as necessary in this strategy as they align with oral function, and the prominence of aesthetics also depends on its role in the patient’s social and psychological health. It is related to the aforementioned *rationalizing strategy* in gerostomatology (Fejérdy, Nagy and Orosz 2007), where the maximal benefit is meant to be attained with the least number of medical interventions. The theoretical groundwork distinguishes these strategies, as the *rationalizing strategy* puts greater emphasis on reducing the number of sessions.

The challenge of this strategy is determining the exact goal of the treatment, as oral function is a concept heavily subjected to each patient’s individual terms of well-being. Eventually this strategy does not adopt a textbook-based definition of oral function but tailors it to the patient’s needs bearing the fundamental principles of beneficence and nonmaleficence in mind. A *functionalist treatment plan* is ethically acceptable for autonomous patients whose own set of values point to sessions limited to restoring function. Likewise, it is also acceptable for patients whose financial, physical, or mental state or other factors compromise their ability to pay more visits to the dentist’s office.

### Preserving Strategy

Following this strategy, the primary aim of dentistry is preserving one’s dental and oral tissues. This can take effect in choosing minimally invasive or microinvasive conservative interventions in treating carious teeth, preferring prostheses which do not require tooth preparation, and attempting periodontic or endodontic treatment for teeth with questionable or lower than questionable prognosis. In this strategy aesthetics rank low in the value hierarchy, and while oral health is a capital value, the intactness of sufficiently functional structures is ranked over maximal function. The management of periodontitis and endodontic treatments demand multiple sessions (Moreira, et al. 2017; Kwon, Lamster, and Levin 2021), and the extraction of the tooth is postponed as far as possible, thus analogously to the idealistic strategy, employing the preserving strategy necessitates numerous visits.

Even though this is an ethically acceptable strategy in most cases if it corresponds to the patient’s own set of values, the shortcoming of this strategy lies in the shortage of human resources, since this strategy frequently requires involving periodontist and endodontist specialists (Barnes, Patel, and Mannocci 2010; Pandya 2019).

### Wish-Fulfilling Strategy

This strategy ranks autonomy a paramount value, usually intertwined with aesthetics. In effect medical interventions are conducted in a professional setting, however the goals of wish-fulfilling procedures disregard maintaining and improving the patient’s health and oral function (Witter, et al. 2020). In this strategy the dentist’s expertise may be submitted to the patient’s own set of values, making the dentist an agent of the patient (Ozar, Sokol, and Patthoff 2018).

In spite of its antagonistic position to other treatment strategies, which each rank certain aspects of oral health ahead of patient autonomy, overlaps in the actual treatment plans may occur even in the case of wish-fulling dentistry. The reason for this is health being defined as biopsychosocial well-being (Larsen 2022), and that oral aesthetics contributes to psychosocial well-being (Kershaw, Newton and Williams 2008; Eli, Bar-Tal and I. Kostovetzki 2001; Feng, Newton and Robinson 2001). Such homology in treatment plans necessarily occur if the impairment is both physical and aesthetic, for example in rehabilitating edentulous patients (Allen and McMillan 2003), although the emphases of the treatment plans may differ. Discord among the *wish-fulfilling strategy* and other strategies becomes apparent to a greater extent if the cosmetic treatment is not restorative and it is performed on functional or semi-functional structures.

A non-exhaustive list of cosmetic dental interventions ranked from least to most invasive, hence from no loss to the complete loss of dental tissue is as follows: bonding dental jewellery on the tooth’s surface, vital tooth bleaching, preparation for veneers, preparation for solo crowns, and tooth extraction for aesthetic restoration. Analogy in ethical considerations in the latter example can be drawn to other medical interventions, in which autonomous patients desire psychosocial benefit despite physical impairment. However, the ethical acceptance of this broad group of procedures varies, as sex-reassignment surgery is commonly estimated to be beneficial to the patient despite causing infertility, while limb-amputation in Body Integrity Identity Disorder is generally not approved of (Kovács 2009). Further on, besides the *wish-fulfilling strategy*, the *functionalist strategy* would also allow impairment of dental hard tissue as long as it is negligible compared to the gains in psychosocial well-being.

Additionally, a utilitarian argument can be made for a shared employment of the wish-fulfilling strategy with other strategies, as granting the patient’s desires upholds the relationship with the patient (Asscher, Bolt, and Schermer 2012). Thus, it may be a tool to gain the patient’s compliance for subsequent medically indicated interventions.

Literature describes a dichotomy between professionalism and commercialism in dentistry, in which wish-fulfilling dentistry would incorporate commercialism (Ozar, Sokol, and Patthoff 2018). However, commerce is not necessarily incompatible with professionalism, and medicine has nearly always been practiced within a marketplace. The ethical employment of this strategy requires the dentist to conform to an extent to the patient’s own set of values, making the dentist an agent of the patient, rather than becoming an actor thriving to fulfil their own interests (Ozar, Sokol, and Patthoff 2018). Furthermore, the author believes that in the occasions, when the practitioner does submit to a treatment plan without medical indication, the fundamental ethical criteria is to minimize risk, for example supporting excellent oral hygiene associated with cosmetic interventions impairing hard dental tissue.

### Acute Treatment Strategy

This strategy focuses on exclusively treating conditions resulting in acute impairment in function, such as acute pain, for example as a result of the exacerbation of chronic inflammation. Usually, an acute treatment strategy seeks to definitively eliminate the cause of the impairment in one session by invasive intervention, or if this is not feasible, it seeks to provide medically indicated curative care in the given session. This strategy resembles gerostomatology’s *postponing strategy* due to the lack of action unless action becomes inevitable (Fejérdy, Nagy, and Orosz 2007).

Despite limiting dental care to acute care cannot typically be the aim of a dental practitioner, notable exceptions exist. These exceptions can be linked to external factors or factors related to the patient. External factors include any form of lack of resources or capacity to perform non acute care, as it is intentionally the case in dental emergency facilities. In such scenarios after the acute intervention is completed, the patient must be referred to another practitioner or recalled to another appointment, where these restraints are eliminated. Factors related to the patient include but are not limited to the following: any patient practicing their autonomy to refuse further dental care, the patient’s treatment posing acute risk to the personnel’s health, e.g., acute contagious infections, however the patient in this example should be recalled to a later appointment, patients with a life-threatening general condition (Status IV. patients according to the American Society Anaesthesiologists’ Physical Status Classification System (Horváth et al. 2021), end-of-life patients, where the stress caused by extensive dental care may exceed its benefit, neglectable oral function, as in comatose patients. In these cases, the benefit and risk of dental care should be evaluated and strategies requiring less intervention should be employed.

### Symptom-Managing Strategy

Similarly to the *functionalist* and *acute treatment strategy*, this strategy also focuses on impaired function. However contrary to them, it is only concerned about impaired function that the patient perceives as such, and as its prime goal is providing non invasive care, it is not interested in eliminating the underlying cause of the impairment. Ultimately this strategy can also be compared to gerostomatology’s postponing strategy (Fejérdy, Nagy, and Orosz 2007), although it does not aim to reduce the number of visits, only their invasiveness. The set of circumstances where this treatment strategy is ethically appropriate largely overlaps with the external and patient-related circumstances described at the *acute treatment strategy*, provided that an acute condition is not present, the maleficence caused even by the acute treatment outweighs the benefit, or an autonomous patient refuses any invasive treatment. In addition to the aforementioned situations, carrying out symptom management instead of invasive dental care is also ethical, if the dentist lacks expertise to perform the adequate intervention, and refers the patient to another professional.

## Discussion

The theoretical framework of the proposed strategies in treatment planning in dentistry is the alternative manifestations and rankings of different aspects surrounding oral health, patient autonomy, invasiveness of interventions, and number of sessions. Further on, the treatment strategies are presented in their relation to the fundamental principles and values in medical and dental ethics, and to the precursor strategies in gerostomatology. According to the complexity of the objectives in the suggested treatment strategies, they can be divided in a category of *extensive treatment strategies* and *specific treatment strategies*.

The category of *extensive treatment strategies* describes those strategies, which intend to improve or preserve the entire oral state of the patient, therefore not limiting the number of sessions. Given there are no external restraints to applying them, or the maleficence of extensive care does not overweigh its benefits, these strategies may always be proposed to the patient, and the treatment plan aligning to the patient’s own values should be selected with respect to patient autonomy. The *idealistic strategy*’s objective is the patient’s rehabilitation to an academically defined morphological and gnathological state. Related to the *idealistic strategy* is the *functionalist strategy*, which aims to achieve the best feasible oral physical and psychosocial function. Despite their different foci, both strategies acknowledge ideal morphology, function, and aesthetics to some degree, since by restoring a severely impaired oral state, the improvement of the morphological state, function, and aesthetics usually occur simultaneously even if the priority differs. The third strategy in this category is the *preserving strategy*, which views preserving one’s own anatomical structures paramount. By pursuing this strategy, the existing level of aesthetics, function, and morphological integrity is maintained but not improved.

In the category of *specific treatment strategies* ordinarily not applicable strategies are outlined because they are less beneficial for the patient. These strategies may focus on a patient’s single condition requiring immediate attention, as in the *acute treatment strategy* or *symptom-managing strategy.* The difference between them is that following former strategy means performing definitive, usually invasive treatment, while the following the latter means performing non definitive, usually non-invasive treatment. Both strategies are ethically appropriate if there are restraints in carrying out more extensive treatment, if an autonomous patient refuses further dental interventions or if carrying out further treatments would severely violate the principle *nil nocere*. The third member of this group is the *wish-fulfilling strategy*, in which the emphasis is on providing commercial dental service according to patient demands, thus regarding patient autonomy paramount. Although the author acknowledges the presence of commerce in medicine, as well the utilitarian argument that fulfilling the patient’s demand may lead the patient to trust their practitioner to perform further medically indicated interventions, the author advises the application of this strategy only if risk is minimized.

During the formation and ethical elaboration of the suggested treatment strategies the author reflected on various possible aims of treatment, such as ideal structure, integrity of existing structures, physical function, psychosocial function, acute care, symptom-relieving care, and serving patient autonomy in correspondence with each other and other ethical values. Thus, the restriction of this article is the scarcity of relevant dental ethical literature. A further limitation of this article is that the ethical arguments are heavily based on the framework created by Beauchamp and Childress. The author acknowledges, that even though the principles of Beauchamp and Childress are often cited, they are not unquestionable, nor suitable for every situation.

The author urges for further research to improve the quantity and quality of available literature on the topic of conflicting values in dentistry with the hopes that this article contributes to this objective.

## Conclusion

This study argues that the aim of a dental treatment plan may be preserving one’s own anatomical structures, achieving ideal morphology, aesthetic outcome, functionality, and immediate relief from symptoms. Additional factors also play a role, such as patient autonomy or considering the number of sessions and how invasive the intervention needs to be. Limitations of this article include the scarcity of pre-existing literature on this topic in dentistry. Further research is necessary to draw a comparison between the aim of treatment plans in general medicine and in dentistry, thus to position dental ethics within medical ethics.

## References

[CR1] Allen, P.F., and A.S. McMillan. 2003. Review of the functional and psychosocial outcomes of edentulousness treated with complete replacement. *Journal of the Canadian Dental Association* 69: 662. 14611716

[CR2] Asscher, E.C.A., I. Bolt, and M. Schermer. 2012. Wish-fulfilling medicine in practice: A qualitative study of physician arguments. *Journal of Medical Ethics* 38: 327–331. 22318414 10.1136/medethics-2011-100103

[CR3] Artun, J. 2002. The role of minimal intervention in orthodontics. *Medical Principles and Practice* 11(S1): 7–15. 12123117 10.1159/000057773

[CR4] Barnes, J.J., S. Patel, and F. Mannocci. 2010. Why do general dental practitioners refer to a specific specialist endodontist in practice? *International Endodontic Journal* 44: 21–32. 20812944 10.1111/j.1365-2591.2010.01791.x

[CR5] Beauchamp, T.L., and J.F. Childress. 1979. *Principles of biomedical ethics*. New York: Oxford University Press.

[CR6] Eli, I., Y. Bar-Tal, and I. Kostovetzki. 2001. At first glance: Social meanings of dental appearance. *Journal of Public Health Dentistry* 61: 150–154. 11603318 10.1111/j.1752-7325.2001.tb03382.x

[CR7] Fejérdy, P., G. Nagy, and M. Orosz. 2007. *Gerosztomatológia az időskor fogászata* [*Gerostomatology is the dentistry of the elderly*]. Budapest: Semmelweis Kiadó.

[CR8] Feng, X.P., J.T. Newton, and P.G. Robinson. 2001. The impact of dental appearance on perceptions of personal characteristics among Chinese people in the United Kingdom. *International Dental Journal* 51: 282–286. 11570543 10.1002/j.1875-595X.2001.tb00839.x

[CR9] Glick, M., D.M. Williams, D. Kleinman, et al. 2016. A new definition for oral health developed by the FDI World Dental Federation opens the door to a universal definition of oral health. *The Journal of the American Dental Association* 147: 915–917. 27886668 10.1016/j.adaj.2016.10.001

[CR10] Horváth, B., B. Kloesel, M.M. Todd, J.C. Cole, and R.C. Prielipp. 2021. The evolution, current value, and future of the American Society of Anesthesiologists physical status classification system. *Anesthesiology* 135: 904–919. 34491303 10.1097/ALN.0000000000003947

[CR11] Kershaw, S., J.T. Newton, and D.M. Williams. 2008. The influence of tooth colour on the perceptions of personal characteristics among female dental patients: Comparisons of unmodified, decayed and “whitened” teeth. *British Dental Journal* 204: E9–E9. 18297050 10.1038/bdj.2008.134

[CR12] Kovács, J. 2006. *A modern orvosi etika alapjai*. Budapest: Medicina Könyvkiadó Zrt.

[CR13] Kovács, J. 2009. Whose identity is it anyway? *American Journal of Bioethics* 9: 44–45. 10.1080/15265160802617886 19132622

[CR14] Kwon, T., I.B. Lamster, and L. Levin. 2021. Current concepts in the management of periodontitis. *International Dental Journal* 71: 462. 34839889 10.1111/idj.12630 PMC9275292

[CR15] Langlois, J.H., L. Kalakanis, A.J. Rubenstein, A. Larson, M. Hallam, and M. Smoot. 2000. Maxims or myths of beauty? A meta-analytic and theoretical review*. Psychological Bulletin* 126: 390–423. 10825783 10.1037/0033-2909.126.3.390

[CR16] Larsen, L.T. 2022. Not merely the absence of disease: A genealogy of the WHO’s positive health definition. *History of the Human Sciences* 35: 111–131. 10.1177/0952695121995355

[CR17] Lussier, J.P., and M. Benigeri. 2008. Survol des caractéristiques de la clientèle des cabinets dentaires et des traitements offerts par les généralistes en 2005 Résultats du sondage de l’ODQ de 2006 [Overview of the characteristics of the clientele of dental practices and the treatments offered by general practitioners in 2005. Results of the 2006 ODq survey—Second part]. *Journal de l’Ordre des dentistes du Québec* 45: 21–24.

[CR18] Moreira, M.S., A.S.N.S. Anuar, T.K. Tedesc, M. Dos Santos, and S. Morimoto. 2017. Endodontic treatment in single and multiple visits: An overview of systematic reviews. *Journal of Endodontics* 43: 864–870. 28457639 10.1016/j.joen.2017.01.021

[CR19] Ozar, D.T., D.L. Schiedermayer, and M. Siegler 1988. Value categories in clinical dental ethics. *The Journal of the American Dental Association* 116(3): 365–368. 3162492 10.14219/jada.archive.1988.0242

[CR20] Ozar, D.T., D.J. Sokol, and D.E. Patthoff. 2018. *Dental ethics at chairside: Professional obligations and practical applications*. Washington, DC: Georgetown University Press.

[CR21] Pandya, N. 2019. The role of a specialist in periodontology. *British Dental Journal* 227(7): 626–627. 31605075 10.1038/s41415-019-0736-2

[CR22] Rule, J.T., and R.M. Veatch. 2004. *Ethical questions in dentistry*. Chicago: Quintessence Pub. Co.

[CR23] Samorodnitzky-Naveh, G.R., S.B. Geiger, and L. Levin. 2007. Patients’ satisfaction with dental esthetics. *The Journal of the American Dental Association* 138(6): 805–808. 17545270 10.14219/jada.archive.2007.0269

[CR24] Spear, F.M., and V.G. Kokich. 2007. A multidisciplinary approach to esthetic dentistry. *Dental Clinics of North America* 51(2): 487–505. 17532924 10.1016/j.cden.2006.12.007

[CR25] Witter, D.J., J.J.J. Kole, G.J. Brands, M.I. MacEntee, and N.H.J. Creugers. 2020. Wish-fulfilling medicine and wish-fulfilling dentistry. *Journal of Dentistry* 96: 103302. 32087260 10.1016/j.jdent.2020.103302

[CR26] World Health Organization. 1980. *International classification of impairments, disabilities and handicaps.* Geneva: WHO.

